# Palliative care and healthcare utilization among metastatic breast cancer patients in U.S. Hospitals

**DOI:** 10.1038/s41598-023-31404-2

**Published:** 2023-03-16

**Authors:** Sun Jung Kim, Isha Patel, Chanhyun Park, Dong Yeong Shin, Jongwha Chang

**Affiliations:** 1 Department of Health Administration and Management, College of Medical Science, Soonchunhyang University, Asan, Republic of Korea; 2 Center for Healthcare Management Science, Soonchunhyang University, Asan, Republic of Korea; 3 Department of Software Convergence, Soonchunhyang University, Asan, Republic of Korea; 4 Department of Health Care Management, Brad D. Smith School of Business, Marshall University, Huntington, WV USA; 5 Health Outcomes and Pharmacy Practice, College of Pharmacy, University of Texas, Austin, TX USA; 6 Department of Public Health Sciences, College of Health, Education and Social Transformation, New Mexico State University, Las Cruces, NM USA; 7 Department of Pharmaceutical Sciences, Irma Lerma Rangel School of Pharmacy, Texas A&M University, College Station, TX 77843 USA; 8 Irma Lerma Rangel School of Pharmacy, Texas A&M University, College Station, TX 77843 USA

**Keywords:** Palliative care, Health policy, Health services

## Abstract

There is a lack of research focused on understanding the different characteristics and healthcare utilization of metastatic breast cancer patients by palliative care use. This study aims to investigate trend of in-patient palliative care and its association with healthcare utilization among hospitalized metastatic breast cancer patients in the US. National Inpatient Sample (NIS) was used to identify nationwide metastatic breast cancer patients (n = 5209, weighted n = 25,961) from 2010 to 2014. We examined the characteristics of the study sample by palliative care and its association with healthcare utilization, measured by discounted hospital charges and length of stay. Multivariable survey regression models were used to identify predictors. Among 26,961 breast cancer patients, 19.0% had palliative care. Percentage of receiving palliative care during the period were gradually increased. Social factors including race, insurance types were also associated with a receipt of palliative care. Survey linear regression results showed that patients with palliative care were associated with 31% lower hospital charges, however, length of stays were not significantly associated. This study found evidence of who was associated with the receipt of palliative care and its relationship with healthcare utilization. This study also emphasizes the importance of receiving palliative care in patients with breast cancer, paving the way for future research into ways to improve palliative care in cancer patients. This study also found social differences and gave evidence of programs that could be used to help vulnerable groups in future health policy decisions.

## Introduction

Breast cancer is the most common cancer among women in the United States. Approximately 13% of women in the U.S. are diagnosed with breast cancer in their lifetime, and it is the cause of death for 3% of American women annually^1^. In 2019, an estimated 286,600 new breast cancer cases and 47,760 deaths were reported^1,2^. Furthermore, the incidence rate for invasive breast cancer has been steadily increasing over the last 15 years^1^. The majority of breast-cancer related deaths are a result of complications from recurrent or metastatic diseases^3^. Metastatic breast cancer is defined as breast cancer that has spread to another part of the body, such as the liver, brain, bones, or lungs^4^. With respect to new breast cancer cases, approximately 6% are metastatic when initially diagnosed^3^. Even when cases are diagnosed in early stages, 30% of these early-stage breast cancer cases will still become metastatic^3^.

Despite significant improvements in treatment, the 5-year relative survival rate for metastatic breast cancer is only 27%, with a median relative survival time of 25 months; significantly less than the 5-year relative survival rate for localized breast cancer of 99%^1,5,6^. The progression of metastatic breast cancer and its different cytotoxic treatments can cause patients to experience increased fatigue, stress, depression, anxiety, and social isolation. These manifestations significantly impact the patient’s psychological well-being and quality of life^7,8^. Metastatic breast cancer remains essentially incurable, and current therapy goals include: the palliation of symptoms, delaying disease progression, improving/maintaining quality of life, and prolonging overall survival^9^. According to a recent guideline by the American Society of Clinical Oncology (ASCO), it is recommended for patients with advanced cancer to receive interdisciplinary palliative care services with concurrent active treatment^10^.

Palliative care is an interprofessional discipline that utilizes a patient-centered approach to address the supportive care needs of patients with advanced cancer, and their families^11^. The clinical importance of palliative care for patients with metastatic breast cancer has been demonstrated in several previous studies that have shown that palliative care facilitates a higher quality of life, less aggressive end of life, lowers depression rate, and maintains or improves survival for patients with metastatic breast cancer^12,13^. Other studies have also demonstrated that integration of palliative care improves patient understanding of the disease, and promotes dialogue that encourages patient involvement in the treatment planning^14^. The European society of Medical Oncology's guidelines strongly recommend introduction of specialty palliative care in metastatic breast cancer, as adequate control of symptoms such as pain should be prioritized^13^. Other clinical benefits are well documented and it is suggested that healthcare professionals recognize their role in the patient's care, as well as end of life integration of palliative care into patient's treatment plans^15,16^.

Previous studies suggested that palliative care was associated with lower costs, and economic benefits of palliative care for metastatic cancer patients were very clear^17–21^ where more end-of life cancer patients are seeking various aggressive treatments^17,18,22^. Administering unnecessary end-of-life care can hinder discussions about proper symptom relief and palliative care. Furthermore, as new anti-cancer treatments are continuously being developed, it is common for palliative care to become delayed for metastatic breast cancer patients until they are very close to end-of-life^15^. In the era of rapid growing cancer patient, understanding of various characteristics for palliative care utilization is very critical^23^, however, evidence of economic benefit regarding with metastatic breast cancer patients have not been reviewed well. There is a lack of previous studies focusing on the aspects of palliative care using a representative population sample^24,25^ and its association with healthcare utilization measured by hospital charges and length of stays (LOS) among metastatic breast cancer patients, it is very crucial to investigate the issue.

Despite numerous studies demonstrating the benefits of palliative care for metastatic breast cancer patients, little is known regarding the pattern of palliative care utilization among metastatic breast cancer patients in the United States. The primary objective of this study is to examine the recent trends of palliative care utilization to identify the various factors associated with the use of palliative care among hospitalized women with metastatic breast cancer. This study will also assess how palliative care was associated with hospital length of stay and hospital charges among women hospitalized with metastatic breast cancer. This study will finally look at these factors and compare them to findings in previous studies on palliative care in other cancers and conditions.

## Materials and method

### Data collection

**Figure 1:**
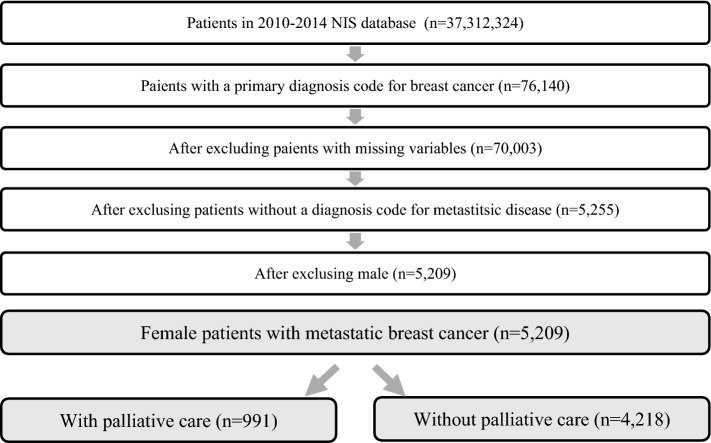
Patient selection process.

The 2010–2014 National Inpatient Sample (NIS) database was used to obtain a population based estimate for women with metastatic breast cancer. NIS data is obtained by Agency for Healthcare Research and Quality (AHRQ)’s Healthcare Cost and Utilization Project (HCUP) and it is not only one of the largest publicly available healthcare dataset but also contains all payer US hospital inpatients records. Among all 2010–2014 NIS sample (N = 37,312,324), as shown in Fig. 1, we first identified primary diagnosis of breast cancer (total n = 76,140) using the International Classification of Diseases, Ninth revision, Clinical Modification (ICD-9-CM) codes for breast cancer. Metastatic stage was determined as the ICD-9 code indicated metastatic disease to other organs (196.0–196.2, 196.5–196.9, 197.0–197.8, 198.80–198.89 and 199.00–199.18). After cleaning the missing other variables, we finally obtained women patients with diagnosis of metastatic stage (N = 5209).

### Variables

The main objective of this study was to investigate the characteristics of palliative care utilization and its association with in-hospital death, length of stay, and hospital charges. The main interesting variable was a receipt of palliative care and we used the ICD-9-CM code of V66.7 in order to identify palliative care for hospitalized patients. The V66.7 code is defined as an encounter for palliative care when hospitalized patients received palliative care consultation. Further, it is used in situations of “palliative care,” “end-of-life care,” “hospice care,” or “terminal care”^26^. Total hospital charges were calculated after adjusting for the annual inflation rate using Centers for Medicare and Medicaid Services estimates^27^. Due to the skewing of distribution for hospital charges and length of stay, we calculated the natural log of those variables. In this study, we adjusted patient-level and hospital-level confounders. Patient level characteristics included: age, race, annual median household income based on ZIP code, primary payer (Medicare, Medicaid, Self-Pay/No Charge, Other, Private insurance), number of comorbidities, severity of illness using all-patient refined diagnosis-related group (APRDRG), and whether patients received surgery, radiation, or chemotherapy. Hospital attributes included information concerning teaching status, location, and bed-size. We defined our variables based on previous literatures using NIS samples^23,28,29^.

### Statistical analysis

To represent the entire population of patients with metastatic stage breast cancer, sampling weights were used for all statistical analyses. We used a sampling weights approach that was employed in previous research^28^. We first examined the characteristics of the final dataset which included patient and hospital characteristics by palliative care use. The patient and hospital characteristics were presented as weighted frequency (percentage) or means (SD). To investigate group differences (whether receiving palliative care), Rao-Scott Chi-Square tests were used for categorical variables.

**Table 1:** Patient and hospital characteristics by palliative care use.

	Total		Palliative care		No palliative care		*p*
	N	Col%	N	Row%	N	Row%	
Unweighted	5209		991		4218		
Weighted	25,961	100%	4934	19.0%	21,028	81.0%	
Age group							
< 40	1448	5.6%	228	15.7%	1220	84.3%	0.249
40–65	14,859	57.2%	2,761	18.6%	12,098	81.4%	
65–80	7108	27.4%	1,418	19.9%	5691	80.1%	
≥ 80	2546	9.8%	527	20.7%	2019	79.3%	
Race							
Black	5571	21.5%	876	15.7%	4695	84.3%	0.034
Hispanic	2457	9.5%	479	19.5%	1978	80.5%	
Asian or Pacific Islander	772	3.0%	148	19.1%	625	80.9%	
Native American/Others	870	3.4%	190	21.8%	681	78.2%	
White	16,291	62.8%	3,242	19.9%	13,049	80.1%	
Median household income							
0–25th percentile	7321	28.2%	1383	18.9%	5937	81.1%	0.284
26th–50th percentile	6498	25.0%	1262	19.4%	5236	80.6%	
51st–75th percentile	5952	22.9%	1030	17.3%	4922	82.7%	
76th–100th percentile	6191	23.8%	1259	20.3%	4932	79.7%	
Primary payer							
Medicare	9796	37.7%	1699	17.3%	8096	82.6%	< 0.001
Medicaid	4485	17.3%	789	17.6%	3696	82.4%	
Self-pay/No charge	1237	4.8%	184	14.9%	1052	85.0%	
Other	826	3.2%	331	40.1%	495	59.9%	
Private insurance	9618	37.0%	1930	20.1%	7688	79.9%	
Number of comorbidities*	2.146	1.818	2.025	1.813	2.174	1.819	0.020
Severity of illness subclass							
APR-DRG 0,1, lowest	4526	17.4%	894	19.8%	3632	80.2%	< 0.001
APR-DRG 2	9018	34.7%	1416	15.7%	7602	84.3%	
APR-DRG 3	9984	38.5%	1889	18.9%	8094	81.1%	
APR-DRG 4, highest	2433	9.4%	733	30.1%	1700	69.9%	
In hospital death							
No	22,135	85.3%	2816	12.7%	19,319	87.3%	< 0.001
Yes	3826	14.7%	2118	55.4%	1708	44.6%	
Surgery							
No	21,154	81.5%	4863	23.0%	16,290	77.0%	< .0001
Yes	4808	18.5%	70	1.5%	4737	98.5%	
Radiation							
No	25,067	96.6%	4771	19.0%	20,296	81.0%	0.790
Yes	894	3.4%	163	18.2%	731	81.8%	
Chemotherapy							
No	24,200	93.2%	4670	19.3%	19,530	80.7%	0.045
Yes	1,761	6.8%	264	15.0%	1498	85.1%	
Hospital status							
Urban nonteaching	9374	36.1%	1798	19.2%	7575	80.8%	0.960
Urban teaching	12,030	46.3%	2281	19.0%	9749	81.0%	
Rural	4557	17.6%	855	18.8%	3703	81.3%	
Region of hospital							
Northeast	5879	22.6%	959	16.3%	4920	83.7%	0.006
Midwest	5181	20.0%	913	17.6%	4268	82.4%	
South	10,194	39.3%	2142	21.0%	8052	79.0%	
West	4708	18.1%	920	19.5%	3787	80.4%	
Bed size of hospital							
Small	6903	26.6%	1438	20.8%	5,465	79.2%	0.129
Medium	12,375	47.7%	2262	18.3%	10,113	81.7%	
Large	6683	25.7%	1234	18.5%	5450	81.6%	
Length of stay (days)*	5.87	6.86	6.46	7.44	5.74	6.71	0.0028
Hospital charge (2010 USD)*	42,754	59,796	37,807	80,564	43,906	53,775	0.0042

*Mean/SD.

**Figure 2:**
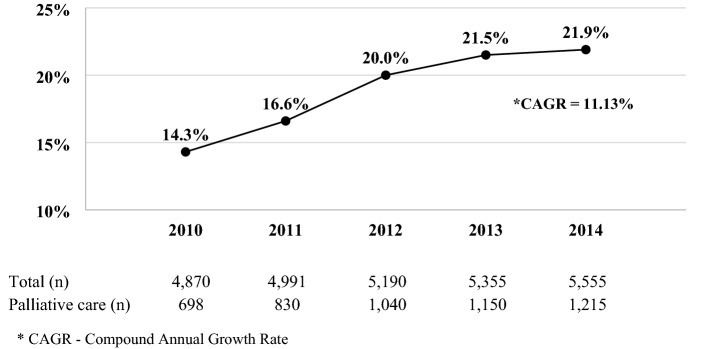
Trend in percentage of receiving palliative care between 2010 and 2014. *CAGR—Compound Annual Growth Rate.

Using the survey logistic regression analysis, the odds ratios and 95% confidence interval (ORs and 95% CI) for receiving palliative care for metastatic breast cancer patients were calculated (Table 2). Via multivariable survey linear regression analysis, the effect of palliative care on both lengths of stay and hospital charges was also examined (Table 3). All analyses were conducted by using SAS statistical software (version 9.4; SAS Institute Inc., Cary, NC, USA). The statistical tests utilized in this study were all two-sided and statistical significance was calculated at *p* value < 0.05.

**Table 2:** Results of Survey Logistic Regression: Odds of receiving palliative care.

Variable	Odds ratios	95% CIs		*P*
Age group				
< 40	Reference			
40–65	1.23	0.85	1.78	0.138
65–80	1.58	1.03	2.43	0.145
≥ 80	1.91	1.19	3.08	0.007
Race				
Black	0.74	0.60	0.93	0.016
Hispanic	1.04	0.78	1.40	0.478
Asian or Pacific Islander	0.93	0.59	1.46	0.876
Native American/Other	1.10	0.76	1.59	0.371
White	Reference			
Median household income				
0–25th percentile	0.82	0.65	1.04	0.521
26th–50th percentile	0.87	0.70	1.09	0.835
51st–75th percentile	0.76	0.61	0.95	0.090
76th–100th percentile	Reference			
Primary payer				
Medicare	0.76	0.59	0.98	0.005
Medicaid	0.96	0.75	1.22	0.481
Self-pay/No charge	0.64	0.42	0.97	0.005
Other	2.46	1.69	3.59	< .0001
Private insurance	Reference			
Number of comorbidities	0.96	0.92	1.01	0.111
Severity of illness subclass				
APR-DRG 0,1, lowest	Reference			
APR-DRG 2	0.83	0.66	1.05	0.234
APR-DRG 3	0.77	0.60	0.97	0.014
APR-DRG 4, highest	1.03	0.74	1.44	0.197
In hospital death				
No	Reference			
Yes	6.59	5.50	7.90	< .0001
Surgery				
No	Reference			
Yes	0.07	0.04	0.12	< .0001
Radiation				
No	Reference			
Yes	1.05	0.70	1.60	0.803
Chemotherapy				
No	Reference			
Yes	0.69	0.50	0.94	0.019
Hospital status				
Urban nonteaching	1.19	0.94	1.50	0.905
Urban teaching	1.38	1.09	1.74	0.007
Rural	Reference			
Region of hospital				
Northeast	0.69	0.54	0.90	0.011
Midwest	0.78	0.60	1.02	0.343
South	0.93	0.74	1.16	0.123
West	Reference			
Bed size of hospital				
Medium	1.18	0.96	1.47	0.107
Large	1.05	0.87	1.27	0.656
Small	Reference

**Table 3:** Results of Survey Linear Regression: LOS and Hospital charges (log transformed).

	Length of stay			Hospital charges		
	Coefficient, β	SE	P	Coefficient, β	SE	*P*
Age group						
< 40	Reference			Reference		
40–65	0.02	0.04	0.545	− 0.10	0.06	0.068
65–80	0.06	0.05	0.191	− 0.22	0.07	0.002
≥ 80	0.07	0.05	0.196	− 0.32	0.08	< .0001
Race						
Black	0.09	0.03	0.001	0.08	0.04	0.021
Hispanic	− 0.03	0.04	0.404	0.11	0.05	0.028
Asian or Pacific Islander	0.07	0.06	0.196	0.11	0.07	0.148
Native American/Other	0.09	0.05	0.079	0.17	0.07	0.012
White	Reference			Reference		
Median household income						
0–25th percentile	0.05	0.03	0.068	− 0.08	0.04	0.030
26th–50th percentile	0.01	0.03	0.919	− 0.13	0.04	0.001
51st–75th percentile	0.02	0.03	0.410	− 0.04	0.04	0.241
76th–100th percentile	Reference			Reference		
Primary payer						
Medicare	0.03	0.03	0.297	0.07	0.04	0.089
Medicaid	0.14	0.03	< .0001	0.11	0.04	0.006
Self-Pay/No Charge	0.05	0.05	0.294	0.01	0.07	0.999
Other	− 0.07	0.05	0.195	− 0.48	0.09	< .0001
Private insurance	Reference			Reference		
Number of Comorbidities	0.05	0.01	< .0001	0.07	0.01	< .0001
Severity of illness subclass						
APR-DRG 0,1, lowest	Reference			Reference		
APR-DRG 2	− 0.15	0.03	< .0001	− 0.23	0.04	< .0001
APR-DRG 3	0.01	0.03	0.950	− 0.09	0.04	0.025
APR-DRG 4, highest	0.25	0.05	< .0001	0.38	0.06	< .0001
In hospital death						
No	Reference			Reference		
Yes	− 0.10	0.03	0.003	− 0.37	0.05	< .0001
Palliative care						
No	Reference			Reference		
Yes	0.05	0.03	0.089	− 0.31	0.04	< .0001
Surgery						
No	Reference			Reference		
Yes	− 0.30	0.03	< .0001	0.29	0.03	< .0001
Radiation						
No	Reference			Reference		
Yes	0.64	0.05	< .0001	0.79	0.06	< .0001
Chemotherapy						
No	Reference			Reference		
Yes	0.62	0.04	< .0001	0.82	0.05	< .0001
Hospital status						
Urban nonteaching	− 0.03	0.03	0.309	0.17	0.04	< .0001
Urban teaching	0.01	0.03	0.915	0.20	0.04	< .0001
Rural	Reference			Reference		
Region of hospital						
Northeast	0.10	0.03	0.001	− 0.09	0.04	0.022
Midwest	− 0.01	0.03	0.649	− 0.45	0.04	< .0001
South	0.05	0.03	0.059	− 0.39	0.04	< .0001
West	Reference			Reference		
Bed size of hospital						
Medium	− 0.01	0.03	0.636	0.04	0.04	0.214
Large	0.00	0.02	0.860	0.11	0.03	< .0001
Small	Reference			Reference		

This study adhered to the Declaration of Helsinki, and the data we used in this study was secondary data in which all patient data were encrypted and unable to identify. This study was approved for waiver from the Institutional Review Board of Soonchunhyang University (202109-SB-083).

## Results

### Patient characteristics

A total 5209 hospital discharges of women with metastatic breast cancer were identified in the 2010–2014 NIS data (weighted n = 25,961) (Fig. 1). Among them, 991 (weighted n = 4934, 19.0%) had palliative care. The general characteristics of patient and hospital characteristics are presented in Table 1. Among metastatic breast cancer patients who died during hospitalization, over half of them received palliative care (55.4%). Among those who did not die, 12.7% received palliative care. The mean LOS and hospital charges were 6.46 days (SD = 7.44 days) and $37,807 (SD = $80,564) for those who received palliative care and 5.74 days (SD = 6.71 days) 43,906 (SD = 53,775) for those who did not receive palliative care.

### Patterns of palliative care use

Figure 2 shows the trends in palliative care use among hospitalized patients with metastatic breast cancer between 2010 and 2014. The rate at which palliative care were increased from 14.3 to 21.9% during the study period (CAGR—Compound Annual Growth Rate = 11.13%, *p* < 0.001).

The ORs of receiving palliative care from multiple regression model is shown in Table 2. Metastatic breast cancer patients who died during their hospitalization were more likely to receive palliative care compared to those who did not die after controlling for all other variables (OR: 6.59, 95% CI: 5.50–7.90). Black patients were associated with fewer odds of using palliative care compared with White; patients with Medicare were less likely to use palliative care, while patients with other types of insurance were associated with higher odds of using palliative care. Additionally, palliative care was significantly less likely used in patients receiving surgery (OR: 0.07, 95% CI: 0.04–0.12) or chemotherapy (OR: 0.69, 95% CI: 0.50–0.94) during the same admission. The use of palliative care was significantly higher in urban teaching hospitals compared to rural hospitals (OR: 1.38, 95% CI: 1.09–1.74).

### Association of palliative care with hospital charges and LOS

The association of hospital palliative care with hospital charges and LOS is shown in Table 3. Receiving palliative care while in the hospital was significantly associated with lower hospital charges (β = − 0.31, *p* < 0.001).The results also revealed that younger age groups, minority populations, and lower income groups were associated with statistically significant higher hospital charges.

## Discussion

This study a retrospective cohort study by selecting metastatic stage breast cancer patients who were hospitalized between 2010 and 2014. Metastatic breast cancer patients that received palliative care increased by 7.6% from 2010 to 2014. The increased utilization of palliative care from year-to-year among metastatic breast cancer patients was consistent with previous American studies on the utilization of palliative care in patients with other medical issues such as chronic obstructive pulmonary disease (COPD)^29^, multiple sclerosis^30^, and a study inclusive of all cancers^11^. Among metastatic breast cancer patients discharged from American hospitals, 19.0% received palliative care, an increase from 14.3% in 2010 to 21.9% in 2014. In comparison to previous studies from 2005 to 2014 that demonstrated 30.0% of patients with COPD received palliative care^29^. The percentage of patients with multiple sclerosis who received palliative care increased from 0.2% in 2005 to 6.1% in 2014^30^. Among all cancer patients, 9.9% received palliative care, and those specifically with advanced stage cancers experienced an increase in palliative care from 3.0% in 2005 to 15.5% in 2014^11^. Rubens et al. found that 4.3% of all breast cancer patients received palliative care; however, their data did not make a distinction between the subjects’ stages of cancer^11^. Though palliative care uptake percentage increased in this study as years progressed, majority of the patients with metastatic breast cancer did not receive palliative care. According to the third edition of the Clinical Practice Guidelines for Quality Palliative Care (NCP guidelines) drafted by the National Consensus Project for Quality Palliative Care in 2013, seriously or terminally ill patients with cancer who are not likely to recover should be considered for palliative care. Palliative care should be delivered by an able interdisciplinary team who can focus on different aspects of a patient’s quality of life and provide care in conjunction with the patient’s and their family’s preferences, beliefs and preferences. Provision of physical comfort, pain and symptom management, psychological assessment, patient education, grief and bereavement services, inclusive healing environment, culturally and linguistically competent care, and coping tools, understanding patient’s social structure and spiritual needs, promoting a peaceful and dignified death and having access to legal and ethical palliative care-based expertise together form the cornerstone of a multi-dimensional palliative care intervention^31^. The NCP guidelines stress the importance of ensuring that in the future, all women with metastatic breast cancer get access to palliative care. Palliative care utilization among metastatic breast cancer patients was significantly higher in urban teaching hospitals, compared to rural hospitals. This is in concurrence with previous studies that have shown that the general practice of palliative care is utilized more often in urban hospitals and teaching hospitals^32,33^.

Type of hospital and geographical location are important factors related to the availability of palliative care. A study examining availability of palliative care in U.S. hospitals found that non-profit hospitals were more likely to have palliative care compared to for-profit hospitals and same holds true for metropolitan areas compared to rural areas^34^. Our study results also showed that a very small number of hospitalized patients undergoing surgery and chemotherapy were less likely to receive palliative care. Patients with metastatic breast cancer have four patterns of clinical progression: smoldering, gradual, rapid and de novo poor condition. Each pattern of progression has an acute and a stable phase. Provision of and the need for palliative care are determined based on the progression status and the phase experienced by the patient^35^. Our study findings highlight a gap in cancer care management and stress the importance of starting palliative care therapy early on, right after metastatic cancer diagnosis, given its beneficial nature as shown by previous research^14^.

Among metastatic breast cancer patients, we identified disparities regarding the receipt of palliative care. Patients with Medicare and black patients were less likely to use palliative care than white patients. Previous studies reveal that lack of awareness, lack of knowledge, fear that may hasten death, and doubt that might cause providers to deliver inadequate care might be associated with lower care for black patients^36,37^. Further, the study suggested the expansion of palliative care programs because this service is underutilized among Medicare beneficiaries^38^. This study also showed that receiving palliative care was associated with lower in-hospital mortality (OR = 6.59), which is consistent with previous studies on different cancers and conditions^11,30^, where palliative care is more likely recommended to patients who are terminally ill or as a last resort^11^. Palliative care has proven to be beneficial for patients with earlier-stage cancers or in combination with traditional treatment^11,29,30^. Many studies regarding this topic show that palliative care is commonly reserved for the elderly. This perpetuates the underutilization of palliative care in patients that can significantly benefit from it. Not having better opportunities to utilize palliative care can subsequently have a negative impact on their health-related quality of life (HRQoL)^39^.

Hospital charges were significantly lowered by $6,099 for metastatic breast cancer patients receiving palliative care, compared to those who were not. The lower hospital charges are consistent with previous studies, which have shown a decrease of $2,261 in MS patients receiving palliative care^30^. COPD patients receiving palliative care experienced a 28.7% decrease in hospital charges, compared to those who did not. Palliative care that is introduced sooner and used in conjunction with treatment has been shown to improve health outcomes and lower hospital charges when compared to palliative care that is introduced later^29^. A study on the use of palliative care in children found that using palliative care was associated with significantly lower overall and daily hospital costs^40^. Mulvey et al. discovered similar results, concluding that patients with head and neck cancer who received palliative care had significantly lower hospital-related charges, but there was no effect on length of stay^39^. In this study, we don’t see statistical significance for length of stay (LOS), which contradicts other studies, such as that of Cheng et al., where it was found that in hospitalized children with cancer, use of palliative care was associated with a shorter length of stay^39^. The study results are mixed depending on the diseases; further study should be conducted to determine how palliative care is associated with LOS.

This study’s findings show the importance of early integration of palliative care and its impact on length of stay, hospital charges. Although there are mixed conclusions surrounding the impact of palliative care on length of stay, it can be inferred that current literature supports the conclusion that palliative care is associated with decreased hospital charges. A small number of studies have focused on palliative care in patients with metastatic breast cancer. This study centers on palliative care utilization in metastatic breast cancer patients and may therefore prove useful in providing insight on the consequences of both late integration, or choosing not to integrate this health-care service. Clinicians providing care to cancer patients and extremely ill patients should evaluate the benefits of palliative care utilization in accordance with patient needs, expectations, and treatment goals^41^. Additional research needs to be conducted on this topic, as its findings have the potential to promote better health outcomes and better experiences for cancer patients.

## Limitations

There are some limitations in our study. A universal ICD-9 V66.7 code is used to identify palliative care services. Hua et al. show that the usage of this code to identify patients that have received palliative care had poor sensitivity and high specificity^26^. Although in patients with metastatic cancer, sensitivity was considerably high (66.3%). Conversely, in patients that died in the hospital, the sensitivity was lower (53.9%). Age should also be considered when using ICD code V66.7, as sensitivity was improved with age restrictions^26^. Validity of the ICD-9 code V66.7 has been shown in other subgroups such as in hospitalized patients with heart failure, but one should evaluate patient characteristics to determine the validity of ICD-9 code in a specific patient population^42^. Also, ICD-9 code V66.7 does not include specific service information such as an expert palliative care team approach or primary physicians prescribing opioids, etc. More research should be done using detailed palliative care information. In this study, we controlled variables for patient and hospital characteristics based on previous literatures. However, confounding factors still exist even though we controlled for patient and hospital characteristics, and it may or may not differentiate the results of this study. Furthermore, our study findings suggest that palliative care patients stayed longer, however, some women with metastatic disease were unpredictable for their survival. Last, due to the nature of NIS data variables, we won’t be able to include very important variables such as chemo given prior to death, ICU admissions, reason for hospitalization etc. for an analysis. Despite these potential limitations due to using an administrative dataset, this study is meaningful to examine nationwide hospital palliative care and its associations with healthcare utilization for breast cancer patients, for which very limited number of studies have been conducted so far. It might provide some insights and will be useful for health policy development, epidemiology research, and/or clinical settings as well.

## Conclusion

This study found evidence that the use of palliative care was significantly associated with decreased hospital charges. This study highlights the impact of receiving palliative care in the breast cancer patients and leads to further studies that identify ways to improve end-of-life care in this patient population. Also, this study found social disparities including race, insurance type, etc. and provide evidence of targeted programs, future health policy consideration for those vulnerable populations.

## Data Availability

All data generated or analysed during this study are included in this published article.
